# Patient preferences for ocular hypertension monitoring: a discrete choice experiment

**DOI:** 10.1136/bmjophth-2024-001639

**Published:** 2024-10-17

**Authors:** Hangjian Wu, Rodolfo Hernández, David P Crabb, Gus Gazzard, Robert A Harper, Anthony King, James E Morgan, Yemisi Takwoingi, Augusto Azuara-Blanco, Verity Watson

**Affiliations:** 1 Health Economics Research Unit, Institute of Applied Health Sciences, University of Aberdeen, Aberdeen, UK; 2 Department of Optometry & Visual Science, City University of London, London, UK; 3 Moorfields Eye Hospital NHS Foundation Trust, London, UK; 4 University College London, London, UK; 5 Manchester Royal Eye Hospital, Manchester, UK; 6 Department of Ophthalmology, Nottingham University Hospitals NHS Trust, Nottingham, UK; 7 Centre for Vision Sciences, School of Optometry, Cardiff University, Cardiff, UK; 8 Department of Applied Health Sciences, University of Birmingham, Birmingham, UK; 9 Centre for Public Health, Queen's University Belfast, Belfast, UK

**Keywords:** Ocular Hypertension, Glaucoma

## Abstract

**Background/aims:**

To elicit the preferences and calculate the willingness to pay (WTP) of patients with ocular hypertension (OHT) for eye monitoring services in the UK.

**Methods:**

Patients with OHT aged at least 18 years recruited from four NHS ophthalmology departments were included in the study. Patients’ preferences and WTP for an OHT monitoring service in the National Health Service were elicited using a discrete choice experiment (DCE) within a postal survey based on six attributes: (1) how OHT monitoring is organised, (2) monitoring frequency, (3) travel time from home, (4) use of a risk calculator for conversion to glaucoma, (5) risk of developing glaucoma in the next 10 years and (6) cost of monitoring. We used a sequential mixed-methods approach to design the survey.

**Results:**

360 patients diagnosed with OHT were recruited with a mean age of 69 years. In the DCE, reducing the risk of conversion to glaucoma was the most important factor influencing respondents’ choice of monitoring service. Respondents preferred hospital-based monitoring services to community optometrist monitoring, and annual monitoring compared with more frequent (every 6 months) and less frequent (every 18 or 24 months) monitoring. These results can be monetised using WTP. Results of heterogeneity analysis suggest that patients with prior experience in community optometrist monitoring preferred this to hospital-based monitoring.

**Conclusions:**

Although hospital-based monitoring is generally preferred, patients with prior experience in community services have a different opinion, suggesting that patients who are unfamiliar with community optometry services may need additional support to accept monitoring in this setting.

**WHAT IS ALREADY KNOWN ON THIS TOPIC**

- Despite the clinical importance of regular monitoring for ocular hypertension—a key risk factor of developing glaucoma, patients’ preferences for ocular hypertension monitoring are rarely discussed.

**WHAT THIS STUDY ADDS**

- Using a discrete choice experiment, we find that reducing the risk of conversion to glaucoma was the most important factor influencing patients’ choice of monitoring service. Patients preferred hospital-based monitoring services to community optometrist monitoring, yet those with prior experience in community optometrist monitoring preferred this to hospital-based monitoring.

**HOW THIS STUDY MIGHT AFFECT RESEARCH, PRACTICE OR POLICY**

- The results suggest that patients who are unfamiliar with community optometry services may need additional support to accept monitoring in this setting.

## Introduction

 Glaucoma is a common chronic eye condition and the second most common cause of blindness in the UK. In the UK, 2% of the population aged 40 years or above is affected by open-angle glaucoma.^1^ Glaucoma is an irreversible disease, but treatment can delay or stop progression. Early detection of disease and regular monitoring are, therefore, vital to reduce the risk of visual impairment or impact of the condition on vision-related quality of life. Elevated intraocular pressure (IOP) is a risk factor for developing glaucoma, and individuals with IOP ≥21 mm Hg without optic nerve damage are diagnosed as having ocular hypertension (OHT). In the UK, OHT prevalence is estimated to be between 3% and 5% for those aged 40 years or above.^2^

Once diagnosed with OHT, individuals are advised to have regular monitoring in a primary or secondary care setting with visual field and/or optic nerve examinations to monitor the possibility of conversion to glaucoma. Pressure-lowering treatment with selective laser trabeculoplasty or medication is then offered to the OHT patients when they are considered to have reached a certain level of future risk or if primary open-angle glaucoma develops.^3^ Across the UK, the monitoring services for OHT patients show considerable variation in terms of type of clinic and frequency of review.^2 4 5^ Generally, patients with a high risk of conversion are monitored in secondary care while lower-risk patients are monitored in community settings when such services exist. Often, after a period of stability without development of glaucoma, patients transfer to less intensive review (fewer tests at lower frequency). ‘Hospital-based virtual clinics’ are a relatively new service model in which patients’ eye tests are carried out by an ophthalmic technician or nurse and reviewed by a clinician who makes a recommendation and writes to patients and their general practitioner with their results.^6^ While National Institute for Health and Care Excellence (NICE) and the Scottish intercollegiate Guidelines Network have published guidelines about referral, discharge, treatment sequence and monitoring frequencies,^2 3^ real-world clinical practice is variable and depends on the capacity and capability of each eyecare unit, the availability of appropriately trained staff and the existence of a community-based service to provide care.

Despite the clinical importance of regular monitoring for OHT patients’ actual review intervals are often greater than recommended. Studies in other settings have found that adherence to monitoring schedules is lower for asymptomatic conditions and monitoring pathways that are poorly aligned with patient preferences.^7 8^ To date, no studies have investigated patients’ preferences for OHT monitoring despite the importance of this information in the design of care pathways to maximise patient adherence. Studies have investigated patients’ preferences for glaucoma monitoring,^69^,^14^ but the generalisability of these results to the OHT population is limited. OHT patients’ awareness of visual disability, treatment burden and consequently their adherence to regular monitoring may differ. In one study, Burr *et al*^1^ explored general population preferences for OHT monitoring using a discrete choice experiment (DCE). However, the general population is unlikely to comprehend the impact of disease monitoring on their life as well as people who experience it.

In this study, we use a DCE survey to explore OHT patients’ preferences for attributes of monitoring services and calculate their willingness to pay (WTP) for those service characteristics. In DCE surveys, respondents are presented with a series of choice tasks that include two or more hypothetical descriptions of, for example, a healthcare service. These services are described by a set of attributes, which vary systematically across the different services. In each task, respondents are asked to choose the service that they most prefer. DCE surveys provide information about the trade-offs that respondents make between a set of attributes specific to a defined healthcare service and have been widely applied in healthcare studies (see Soekhai *et al*^15^ for a review of DCE applications in healthcare). This approach and similar conjoint analysis experiments have previously been used to elicit glaucoma patient preferences.^9 11 16 17^

## Materials and methods

### Study sample

The multicentre study included four (UK) NHS sites (Belfast Health and Social Care Trust, Nottingham University Hospitals Trust, Manchester University NHS Foundation Trust and London Moorfields Eye Hospital NHS Foundation Trust). OHT patients who had been under active hospital review within the past year were identified by clinicians or research nurses from medical records. The inclusion criteria were a diagnosis of OHT by a health professional and aged 18 years or above. Based on the recommended sample sizes for DCE studies,^18^ our target sample size was 375 respondents.

### Data collection

Between June and September 2023, the study pack was posted to potential study participants. The study pack included a personalised invitation letter, participant information sheet, the DCE survey for self-completion and a prepaid return envelope. Postcard reminders were sent to all potential respondents 1 week after the initial mailout. 1250 potential respondents were identified across the sites based on an assumed response rate of 30%.^19^ Implied consent was assumed if a participant completed and returned the survey. The survey asked respondents to complete 10 DCE choice tasks and describe their current OHT monitoring and socioeconomic status.

### The discrete choice experiment

Following best practice recommendations for the development of DCEs, the attributes and levels included in the DCE were chosen following a literature review and qualitative research with OHT patients.^18 20^ Six attributes were included to describe alternative OHT monitoring services (table 1): how OHT monitoring is organised, visit frequency (every 6–24 months), travel time from home (15–60 min), clinicians use of a risk calculator to inform the monitoring plan (no, yes), risk of developing glaucoma in 10 years (5%–20%) and monitoring cost (£40–£240). The cost was described as all out-of-pocket costs associated with attending a monitoring service for the next 2 years (eg, transportation and loss of earnings due to attending an appointment). Please see online supplemental material 1 for further details about the DCE development.

**Table 1:** Discrete choice experiment attributes and levels

Attributes	Levels				
Where you go to have your tests tested and who carries out the tests	Face to face hospital service: Your eyes will be tested by an eye doctor at the hospital. The eye doctor will talk with you about your tests, current situation and follow-up plans on the day of the test. (FACETOFACE)*	Hospital-based virtual clinic: Your eyes will be tested by a technician at the hospital. After the tests, an eye doctor will review your test results. You will receive a letter about your tests, current situation and follow-up plans another day. (VIRTUAL)	Community optometrist: Your eyes will be tested by a local optometrist. The optometrist will talk with you about your tests, current situation and follow-up plans on the day of the test.		
How often your eyes are tested(FREQUENCY)*	Once every 6months	Once every 12months	Once every 18months	Once every 24 months	
Travel time from home(TRAVEL)	15 min	30 min	45 min	60 min	
This eye service plan is based on… (CALCULATOR)	Eye doctor’s experience	Eye doctor’s experience and the results of a risk calculator			
Out of 100 people with ocular hypertension how many will convert to glaucoma in the next 10 years (RISK)	5	10	15	20	25 (no further visits)
Your cost of attending eye care services over the next 2 years (*COST*)	£40	£100	£180	£240	£0 (no further visits)

* Corresponding variable names used in the statistical analysis.

**Figure 1:**
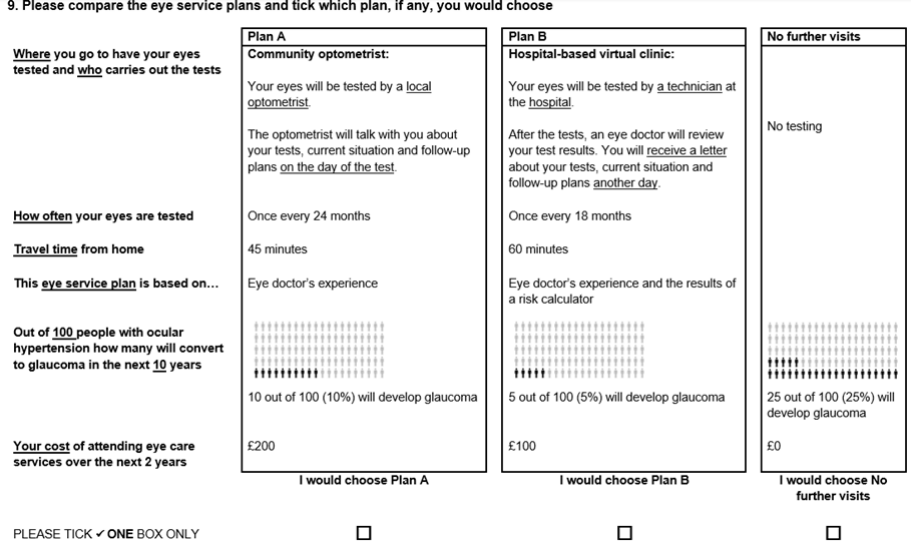
An example of the choice tasks used discrete choice experiment in the questionnaire.

Based on the attributes and levels, a D-efficient fractional-factorial design was used to construct 20 choice tasks using the Ngene software (V.1.3.0).^21^ A D-efficient design minimises the error variance of the estimator used for analysis. This means that the analysis model and any prior estimates must be specified at the design stage. The analysis model was specified as main effects only and given the lack of strong evidence regarding the direction of the parameters, all the parameter priors were assumed to be zero. The design aimed to select 20 choice tasks, which were split into 2 blocks of 10 tasks to reduce the burden on respondents. This design meant that there were 2 versions of the survey each with 10 choice tasks. Respondents were asked to imagine that they were offered a chance to change their OHT monitoring service. In each choice task, respondents were asked to ‘choose’ the monitoring service that they would prefer from two alternative services A or B, or ‘no further visits’ (figure 1). Patients were involved in the design and conduct of our research. The survey was tested in a series of four ‘think-aloud’ interviews with patient representatives and, to ensure the survey was clear and readily understood, minor wording changes were made.

### Analyses

The DCE data were modelled using a random utility framework. This framework assumes that the utility *U* a respondent *n* receives from monitoring service *i* in choice task *t* can be represented by a deterministic and a random part^22^:

(1) $$U_{itn}=\beta X_{itn}+\varepsilon_{itn}$$

$X_{itn}$ is a vector representing the DCE attributes presented in alternative *i,* β is the marginal utilities and $\varepsilon_{itn}$ is the error term following a Gumbel distribution. We assume that respondents choose the monitoring plan that brings them the highest utility in each choice set. Equation 1 can be rewritten as:

(2) $$\begin{array}{ll} U_{itn}= & ASC_{0}+\beta_{1}FACETOFACE+\;\beta_{1}VIRTUAL+\beta_{3}FREQUENCY\_6+\\ \beta_{4}FREQUENCY\_12+\;\beta_{5}FREQUENCY\_18+\beta_{6}TRAVEL+\\ \beta_{7}CALCULATOR+\beta_{8}RISK+\beta_{9}COST+\gamma_{n}+\varepsilon_{itn} \end{array}$$

$$\gamma_{n}∼N\left(0,\sigma \right)$$

where ${ASC}_{0}$ represents an alternative-specific constant for the monitoring alternatives and captures respondents’ preferences to be monitored or not. The βcoefficients represent the mean marginal utility of each attribute on the utility of a monitoring service. The interpretation of the mean marginal utilities depends on the unit of measurement. FACETOFACE and VIRTUAL represent the organisation attribute and were coded as dummy variables that measure the mean marginal utility of a face-to-face hospital service and a hospital-based virtual clinic relative to a community optometrist. The FREQUENCY of testing was coded as dummy variables to allow for non-linearities in respondents’ preferences for monitoring frequency and these measured the mean marginal utility of a 6-month (FREQUENCY_6), 12-month (FREQUENCY_12), 18-month (FREQUENCY_18), monitoring interval relative to a 24-month interval. *TRAVEL* was a linear variable and represented the mean marginal utility of a 1-hour increase in travel time. CALCULATOR was a dummy variable that measured the mean marginal utility of the monitoring interval being based on the ‘eye doctor’s experience and the results of a risk calculator’ compared with ‘eye doctor’s experience’ alone. RISK was linear and measured the 5-year risk of developing glaucoma. COST was a linear and continuous variable that represented the effect of a £1 increase in the cost of attending eye care services over the next 2 years. We expected that respondents would prefer shorter travel times and lower monitoring service cost but had no a priori preference expectations for the remaining attributes. We also calculated respondents’ mean marginal WTP for change in OHT monitoring service^18^,^20^ which allowed us to compare respondents’ values across the attributes. The marginal WTP for a service attribute X is given by the ratio of the coefficient of the service attribute to the coefficient of the cost attribute:

(3) $$WTP_{X}=-\frac{\beta_{x}}{\beta_{9}}$$

We used the delta method to construct 95% CIs for the estimated WTP.^23^

We explored observed preference heterogeneity by interacting selected respondent characteristics with the DCE attributes. First, we tested the effect of previous OHT monitoring experience on preferences for monitoring attributes. We explored interactions between respondents’ previous and recently experienced OHT monitoring organisation and the DCE attributes. We explored interactions between selected socioeconomic variables such as age and sex with DCE attributes.

All data analyses were performed by using STATA V.17.0.^24^ The DCE data were analysed using an error component logit (ECL) model to account for potential correlation across respondents’ choice tasks.^25^ Therefore, the additional error term ($\gamma_{n}$) captured any individual-specific error and was specified to follow a standard normal distribution. The model was estimated using simulated maximum likelihood estimation with 1000 Halton draws.^26^

## Results

### Sample characteristics

360 surveys were returned (response rate=29%) of which 99.2% fully or partially completed the DCE choice tasks (ie, 357; 3 did not complete the DCE section at all). Respondents who failed to complete any of the choice tasks were excluded from the analysis.

Online supplemental table A.1 summarises the respondents’ characteristics. The mean age was 68.6 years (SD=11.2), with 54.2% male and 63.5% retired. Most respondents were diagnosed with OHT more than 5 years ago (60.2%). Almost all respondents had experience with a face-to-face hospital service (91.5%). Fewer respondents had experience with hospital-based virtual clinics (42.5%) or community optometrist monitoring (47.3%). The frequency of monitoring varied across respondents with 59.5% of respondents having a 12-month or longer monitoring interval and 40.5% having a 6-month or shorter interval.

### Patient preferences

Table 2 presents the results of the ECL model. The positive and significant alternative-specific constant (14.519; 95% CI=(0.194, 28.845)) suggests that respondents prefer to have their OHT monitored. Respondents preferred face-to-face hospital service (β=0.516; 95% CI=(0.358, 0.675)) and hospital-based virtual clinic monitoring (β=0.225; 95% CI=(0.073, 0.377)) compared with monitoring by a community optometrist. Respondents preferred more frequent monitoring with the monitoring intervals of 6, 12 or 18 months compared with 24 months (β=0.667 (95% CI= (0.499, 0.835)), β=0.869 (95% CI=(0.692, 1.047)), β=0.284 (95% CI=(0.148, 0.420)), respectively). The most popular monitoring interval was 12 months. Respondents preferred shorter travel times to the monitoring location (β=−0.233; 95% CI=(−0.410, −0.056)). Respondents had no preference for a monitoring plan based on the results of the risk calculator to be used in addition to the eye doctor’s experience. As expected, respondents preferred a lower risk of developing glaucoma in the next 10 years (β=−0.981; 95% CI=(−1.127, −0.834)) and that the cost of the monitoring service was lower (β=−0.002; 95% CI=(−0.002, −0.001)).

**Table 2:** Patients’ preferences for the attributes of eye monitoring services

Attribute	Coefficient	95% CI	P value
Estimated preferences			
Alternative-specific constant (mean)	14.519	0.194, 28.845	0.047
Alternative-specific constant (SD)	6.943	−1.145, 15.032	0.092
How monitoring is organised			
Community optometrist	Reference		
Face-to-face hospital service	0.516	0.358, 0.675	<0.01
Hospital-based virtual clinic	0.225	0.073, 0.377	<0.01
Frequency of eye tests			
Every 24 months	Reference		
Every 18 months	0.284	0.148, 0.420	<0.01
Every 12 months	0.869	0.692, 1.047	<0.01
Every 6 months	0.667	0.499, 0.835	<0.01
Travel time from home	−0.233	−0.410, −0.056	0.01
Risk calculator			
Eye doctor’s experience only	Reference		
Eye doctor’s experience and a risk calculator	0.0411	−0.030, 0.112	0.259
Risk of developing glaucoma	−0.981	−1.127, −0.834	<0.01
Cost of attending eye care services	−0.002	−0.002, −0.001	<0.01
Model information			
Log likelihood	−2099		
AIC	4220		
Number of participants	357		
Number of parameters	11		
Number of observations/choices	3264		

Risk of developing glaucoma is rescaled by 0.1.

AICAkaike information criterion

**Figure 2:**
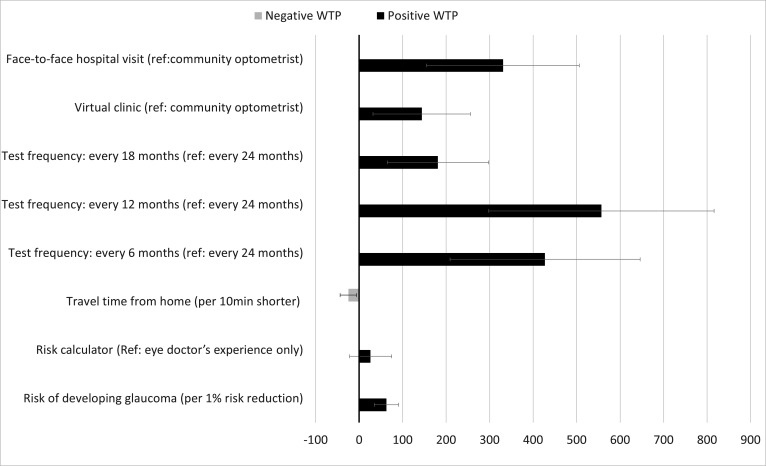
The column on the left of the graph lists the labels for the attribute levels, with the reference levels stated in parentheses. The black bars indicate positive WTP estimates (in GBP) and grey bars indicate negative WTP estimates (in GBP). CIs for the WTPs are shown. WTP, willingness to pay.

Figure 2 presents the mean WTP estimates and 95% CIs for unit changes in the DCE attributes. Respondents most strongly supported monitoring services that are more expensive for healthcare services to provide but that reduced their risk of conversion to glaucoma more. Respondents are willing to pay £628 (95% CI=(£348, £908)) over the next 2 years for a monitoring service that reduces their risk of conversion by 10 percentage-points. Respondents were willing to pay £557 (95% CI=(£298, £816)) more over the next 2 years for a monitoring service with tests every 12 months compared with every 24 months. However, they were willing to pay £130 (95%=(£89, £170)) less for a shorter test interval, that is, every 6 months. Respondents preferred face-to-face hospital monitoring by an eye doctor or a hospital-based virtual clinic rather than community optometrist (£331 (95% CI=(£155, £506)) vs £144 (95% CI=(£32, £256)) over 2 years, respectively).

**Figure 3:**
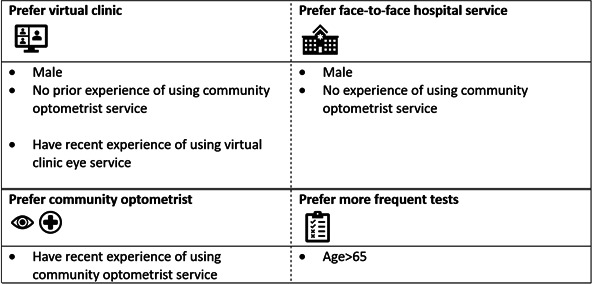
We find that preferences for selected attributes (virtual clinic, face-to-face hospital services, community services and test frequency) differ according to several patient characteristics (sex, experience and age). For example, results for virtual clinic at the top-left corner suggest that male, those who have no prior experience of using community optometric services and those who have recent experience of using virtual clinic eye services have higher preference for virtual clinic than those who are female, who have prior experience of using community optometric services and those who have no recent experience of using virtual clinic eye services. Age >65 is a dummy variable which equals to 1 if a patient’s age is over 65 and equals to 0 otherwise; Male is a dummy variable which equals to 1 if a patient is male and equals to 0 if females; Prior/recent experience of using community optometrist service (virtual clinical eye service) is a dummy variable which equals to 1 if patients self-reported that they had used community optometrist service (virtual clinical eye service) before, or during the last eyecare visit, and equals to 0 otherwise.

The results of the observed preference heterogeneity analysis are summarised in figure 3, which reports the attributes for which we found statistically significant interactions. The full results of the interaction analysis are shown in online supplemental table A.2. We found that male respondents preferred hospital-based virtual clinics more than female respondents (p=0.000). Respondents’ prior experience of monitoring impacts their preferences. Respondents who had never experienced monitoring by a community optometrist preferred hospital-based services (face-to-face hospital monitoring by an eye doctor (p=0.000) or a hospital-based virtual clinic (p=0.000) compared with those who had experienced monitoring by a community optometrist. Furthermore, those who previously experienced monitoring by a community optometrist preferred this to either hospital-based service (p=0.003 for the face-to-face hospital monitoring and p=0.004 for hospital-based virtual clinic).

## Discussion

This paper is the first to estimate OHT patients’ preferences for monitoring services in the UK. We used a DCE to understand patients’ preferences for attributes of monitoring. Previous studies in this area sought the views of glaucoma patients or the general population. The results indicate that the organisation of the review process, the type of health professional involved in testing, test frequency, travel time, risk of developing glaucoma and cost of service all influenced patients’ choices of OHT monitoring. Our results also showed that patients were willing to trade-off different service attributes and cost of the services, which allowed us to calculate their WTP for different attributes of these services.

The latest NICE (UK) guidelines recommend variable risk-based monitoring intervals, between every 1 and 24 months depending on a patient’s risk profile, certainty of test results and control of IOP.^2^ Our results reveal that patients’ preferred monitoring interval is 12 months compared with 6-monthly intervals or greater. Similar to Burr *et al*,^1^ we found that the aspect of monitoring most valued was the reduction in the risk of converting to glaucoma. However, our results suggest that the organisation of the monitoring process is also important to patients, in contrast to Burr *et al*’s observation that this attribute was not important to a general population sample^1^ In this study, the organisation attribute combines the monitoring location and health professionals involved. Our results suggest that patients preferred monitoring by more senior health professionals (an eye doctor in the hospital compared with an optometrist) which is consistent with similar studies investigating patient preferences for glaucoma monitoring.^9 11^ Shorter travel times were preferred by respondents—similar to findings reported by Bhargava *et al*^9^ and Muth *et al*^10^ in studies of glaucoma patients.

Our results also show the importance of preference heterogeneity analysis in revealing patients’ preferences for regular eye monitoring. We found that, on average, respondents preferred both face-to-face hospital monitoring by an eye doctor and a hospital-based virtual clinic to community optometrist monitoring. However, patients with prior experience in community optometrist monitoring preferred this to hospital-based monitoring. The existence of preference heterogeneity based on respondents’ experience suggests that patients who are unfamiliar with community optometry services may need additional support to accept monitoring in this setting. In addition, it indicates that once patients have experienced community-based monitoring they are generally comfortable with it and prefer it, this is an important consideration in future development of OHT monitoring pathways.

Our analysis has three limitations. First, in the DCE the monitoring frequency ranges from every 6 to 24 months. These options represent the suggested intervals for patients with low to medium risk of developing glaucoma. Some high-risk patients may have shorter intervals (eg, every 3 months) and as such, some respondents may find the longer intervals unrealistic. However, these patients accounted for a small percentage of the OHT population. Second, in the survey design stage, we combined several features (eg, healthcare professional, place of testing and testing environment) into one attribute—the organisation; this reflects current service organisation in the UK NHS and the realistic constraints of staffing availability and location and reflects how participants in the qualitative research discuss their monitoring (eg, doctor–patient communication, diagnostic accuracy). However, the integrated attribute means that we cannot separate preferences for several features (eg, location and staffing). Future research would be required to disentangle these elements to better understand patients’ preferences for these aspects of care. Third, WTP values for some attributes of services are higher than the highest level of cost (£240) presented in the DCE. This result can be attributed to the issue of WTP overshooting or cost non-attendance.^27^ One reason could be that respondents were willing to pay more for monitoring services than the highest cost presented in the DCE. High WTP (due to low impact of cost on utility) could also be an indication of protest to paying.^28^

## Conclusion

This study contributes to the understanding of OHT patients’ preferences for service attributes of regular eye monitoring. To our knowledge, it is the first study conducted using a large sample of OHT patients. Reducing the risk of conversion to glaucoma is the most important factor influencing respondents’ choice of monitoring service. The organisation of monitoring is also important to respondents, and the preference can differ depending on their prior experience with hospital or community-based health providers.

## supplementary material

10.1136/bmjophth-2024-001639 online supplemental file 1

## Data Availability

Data are available on reasonable request.
