# Supplementary material for: Patient preferences for ocular hypertension monitoring: a discrete choice experiment
Source: BMJ Open Ophthalmol. 2024 Oct 17;9(1):e001639. doi: 10.1136/bmjophth-2024-001639 (PMC11603805; doi:10.1136/bmjophth-2024-001639)
Supplement: online supplemental file 1 [file bmjophth-9-1-s001.pdf]

Supplementary Material 1: Qualitative research to inform the design of the Discrete Choice Experiment

Best practice recommendations for the design and analysis of discrete choice experiments (DCEs) advises that the attributes and levels are informed by qualitative research with the target population[1].

Methods

We undertook three online bulletin boards (OBBs) with people with OHT in the UK. The aim of the OBBs was to understand individuals' experience of OHT monitoring and their preferences for alternative monitoring services. The OBB is a form of asynchronous online focus group which can reach a broader population [2,3] and recruit many participants quickly [4–6]. Research shows that online focus groups and face-to-face focus groups lead to comparable results [7,8]. An advantage of an asynchronous OBB is that participants can sign in and contribute to the on-going discussion at a time that suits them and for as long or short a time period as they wish.

Each OBB was live for one week from a Monday morning and accessible to participants for 24 hours a day. On each weekday that the OBB is live, one question was posted by a moderator. The questions asked about individuals' experience of OHT monitoring services and were open-ended to encourage expansive answers. Participants could respond to the daily question, questions from previous days and the comments of other participants.

SurveyEngine, a survey company, recruited individuals from the UK general public who met the inclusion criteria: People who are 18 years or older, members of the general public who are living in the UK, Individuals with OHT or early-stage glaucoma as diagnosed by a health professional in the UK, being able to access the internet and use the OBB. Ethics approval was granted by the School of Medicine, Medical Sciences and Nutrition Ethics Review Board, University of Aberdeen. Approval number: SERB 2022/5/2390.

The OBBs were moderated by the research team. The record of the discussion was downloaded as a text file and the comments provided by respondents treated as if they were verbatim statements in a focus group. The transcripts of the OBB were analysed using inductive thematic analysis. Two researchers independently read the transcripts and coded the statements. These coded statements were then grouped into themes following a discussion between three researchers.

Results: 23 people with OHT in the UK were recruited to the OBBs. The themes that emerged included differences in OHT monitoring experienced by participants, travel convenience, monitoring frequency and risk of converting to glaucoma. The research team discussed the themes in meetings and selected potential attributes for the DCE. Six attributes were selected to represent the characteristics of alternative OHT monitoring services (Table 1): how OHT monitoring is organised, visit frequency (from every 6 to every 24 months), travel time from home (from 15 to 60 minutes), clinicians use of a risk calculator to inform the monitoring plan (no, yes), risk of developing glaucoma in 10 years (from 5% to 20%) and monitoring cost (from £40 to £240).

The organisation of OHT monitoring differed across OBB participants. The main differences were characterised by the health care professional who carried out the monitoring and reviewed the test outcomes (and related to this, the location) and how information was provided to patients. Consequently, for this attribute we selected levels to capture the range of plausible services that could be offered within an NHS setting. The interval until the next appointment is often recommended by the health professionals during each clinical visit based on patients' risk disease progression. However, this interval can also be adjusted according to patients' preferences, as clinicians' views are not always aligned with the views of patients [9]. Frequency of tests has been found to be important element of glaucoma monitoring [9,10]. Related to visit frequency, travel time (from home) has also been influential to eyecare services in the literature [9,11,12] and from the results of our OBB. For example, when

participants were asked to describe their views about how often eye tests should be organised, one participant in the OBB said:

> *"The check ups for me seem to be a lot more haphazard than the old days and if you have to wait 18 months or more between visits it's a real worry as you have no idea what's going on." (Participant 004, Day3, Wave 1)*

Risk of developing glaucoma was an important reason why OHT patients in the OBB attended their current monitoring. The levels of risk of conversion were informed by the literature [13], as well as clinical experts in the research team.

The NHS has recommended taking digital transformation into account when determining disease diagnosis and management in a long-term plan [14]. A risk calculator, also known as risk prediction tool, is a digital aid supporting health professionals' decision making on monitoring plan of OHT patients. The risk calculator computes and categorises a patient's risk of developing glaucoma based on several patient characteristics, such as age, intra-ocular pressure and family history of glaucoma using a scientifically validated algorithm, which is then used by the health care team to inform decision making on personalised monitoring plan, e.g., frequency of monitoring. Research on the development of risk prediction tool has been recommended as a key research topic by NICE [15]. Given the potential for a risk prediction calculator to be used in clinical practice, it is important to understand how patients view this new digital tool.

A cost of monitoring attribute was included to allow us to calculate respondents' willingness to pay (WTP) for OHT monitoring services. This was described as all out-of-pocket costs associated with attending a monitoring service for the next two years. Whilst OHT monitoring is provided free at the point of use in the NHS, OBB participants reported that they incurred out of pocket costs associated

with attending OHT monitoring appointments related to travel costs, and loss of earnings. Given that the frequency of testing varied across difference service descriptions, we describe the costs over a two-year period to allow the costs of each service to be easily compared.

Supplementary material 2: additional results

Table A.1: Summary statistics of individual characteristics

Variables	N	Statistic
Age (years), mean ± SD	343	68.6±11.2
Male, n (%)	343	186 (54.2)
Retired, n (%)	342	217 (63.5)
Diagnosed with OHT in more than 5 years, n (%)	352	212 (60.2)
Experience of eyecare services, n (%)		
Face-to-face hospital visit	353	323 (91.5)
Hospital-based virtual clinic	353	150 (42.5)
Community optometrist	353	167 (47.3)
Eye tests frequency equal or longer than annually, n (%)	341	203 (59.5)

N- number in sample; SD = standard deviation.

Table A.2: Preference estimation with interaction effects

	Age>65	Male	With prior experience of community-based eye services	With prior experience of virtual eye clinics	With recent experience of community-based eye services	With recent experience of virtual eye clinics
**Mean effects**						
Face-to-face hospital visit	0.235* [-0.017,0.487]	0.147 [-0.065,0.360]	0.725*** [0.518,0.931]	0.433*** [0.230,0.635]	0.671*** [0.493,0.849]	0.357*** [0.184,0.530]
Hospital-based virtual clinic	-0.014 [-0.261,0.232]	-0.118 [-0.335,0.099]	0.527*** [0.328,0.726]	0.100 [-0.094,0.294]	0.422*** [0.247,0.596]	0.054 [-0.114,0.223]
Frequency of eye tests (linear) [1]	-0.199** [-0.382,-0.017]	-0.493*** [-0.659,-0.327]	-0.430*** [-0.586,-0.273]	-0.350*** [-0.484,-0.216]	-0.486*** [-0.622,-0.349]	-0.407*** [-0.532,-0.282]
Travel time from home	-0.079 [-0.402,0.243]	-0.064 [-0.305,0.177]	0.135 [-0.090,0.361]	-0.001 [-0.216,0.214]	0.025 [-0.162,0.212]	-0.051 [-0.234,0.132]
Risk calculator (Ref: eye doctor's experience only)	0.056 [-0.058,0.169]	0.142*** [0.055,0.230]	0.095** [0.009,0.180]	0.077** [0.006,0.150]	0.080** [0.005,0.155]	0.062* [-0.005,0.129]
Risk of developing glaucoma	-0.975*** [-1.205,-0.744]	-0.836*** [-1.036,-0.636]	-0.864*** [-1.025,-0.703]	-0.856*** [-1.025,-0.687]	-0.884*** [-1.029,-0.739]	-0.881*** [-1.033,-0.728]
Cost of attending eye care services	-0.002*** [-0.003,-0.000]	-0.001 [-0.002,0.000]	-0.000 [-0.001,0.001]	-0.001** [-0.002,-0.000]	-0.001** [-0.002,-0.000]	-0.001** [-0.002,-0.000]

Table A.2: continued

	Age>65	Male	With prior experience of community-based eye services	With prior experience of virtual eye clinics	With recent experience of community-based eye services	With recent experience of virtual eye clinics
Alternative specific constant (mean)	13.11* [-0.752,26.965]	16.34*** [5.453,27.220]	8.199*** [2.939,13.459]	14.388*** [5.543,23.234]	13.938*** [5.322,22.553]	14.345*** [4.409,24.280]
Alternative specific constant (SD)	6.755* [-0.725,14.235]	6.339 *** [2.170,10.508]	3.553*** [1.194,5.913]	6.134*** [1.709,10.560]	6.313*** [2.268,10.358]	6.244*** [1.339,11.148]
**Interaction effects**						
Face-to-face hospital visit	0.222 [-0.088,0.533]	0.392** [0.093,0.690]	-0.705*** [-0.998,-0.410]	-0.103 [-0.400,0.194]	-1.059*** [-1.368,-0.750]	0.102 [-0.242,0.446]
Hospital-based virtual clinic	0.280* [-0.025,0.584]	0.548*** [0.250,0.845]	-0.688*** [-0.978,-0.399]	0.222 [-0.073,0.517]	-0.802*** [-1.113,-0.491]	0.557*** [0.229,0.886]
Frequency of eye tests (linear) [1]	-0.345*** [-0.574,-0.117]	0.124 [-0.105,0.353]	-0.009 [-0.235,0.217]	-0.213* [-0.446,0.021]	0.150 [-0.101,0.401]	-0.158 [-0.443,0.127]
Travel time from home	0.042 [-0.327,0.411]	0.028 [-0.299,0.356]	-0.327** [-0.641,-0.013]	-0.092 [-0.410,0.225]	-0.298 [-0.662,0.068]	-0.075 [-0.451,0.300]

Table A.2: continued

	Age>65	Male	With prior experience of community-based eye services	With prior experience of virtual eye clinics	With recent experience of community-based eye services	With recent experience of virtual eye clinics
Risk calculator (Ref: eye doctor's experience only)	0.035<br>[-0.099,0.169]	-0.107*<br>[-0.231,0.017]	-0.016<br>[-0.139,0.107]	-0.002<br>[-0.128,0.124]	0.025<br>[-0.113,0.164]	0.066<br>[-0.086,0.218]
Risk of developing glaucoma	0.128<br>[-0.149,0.405]	-0.127<br>[-0.395,0.141]	-0.049<br>[-0.313,0.215]	-0.063<br>[-0.323,0.195]	-0.060<br>[-0.389,0.269]	-0.039<br>[-0.325,0.245]
Cost of attending eye care services	0.001<br>[-0.001,0.003]	-0.000<br>[-0.002,0.001]	-0.001*<br>[-0.003,0.000]	-0.000<br>[-0.002,0.001]	-0.001<br>[-0.003,0.001]	0.001<br>[-0.001,0.002]
Alternative specific constant (mean)	3.587<br>[-1.967,9.141]	-3.391<br>[-7.564,0.781]	3.685<br>[-2.705,10.075]	-0.750<br>[-4.676,3.177]	1.433<br>[-2.070,4.937]	-0.439<br>[-3.902,3.024]
N	3,264	3,129	3,234	3,234	3,185	3,185
AIC	4250	4070	4190	4228	4095	4157

(1) lower coefficient of test frequency (in linear form) means preferences for more frequent tests
Standard errors in parentheses * p < .10, ** p < .05, *** p < .01
AIC = Akaike Information Criterion

References

1. Coast J, Al-Janabi H, Sutton EJ, et al. Using qualitative methods for attribute development for discrete choice experiments: issues and recommendations. *Health Econ*. 2012;21(6):730-741. doi:10.1002/HEC.1739
2. Guise J, Widdicombe S, McKinlay A. “What is it like to have ME?”: the discursive construction of ME in computer-mediated communication and face-to-face interaction. *Health (London)*. 2007;11(1):87-108. doi:10.1177/1363459307070806
3. Kenny AJ. Interaction in cyberspace: an online focus group. *J Adv Nurs*. 2005;49(4):414-422. doi:10.1111/J.1365-2648.2004.03305.X
4. Moloney MF, Dietrich AS, Strickland O, Myerburg S. Using internet discussion boards as virtual focus groups. *Advances in Nursing Science*. 2003;26(4):274-286. doi:10.1097/00012272-200310000-00005
5. Woodyatt CR, Finneran CA, Stephenson R. In-Person Versus Online Focus Group Discussions: A Comparative Analysis of Data Quality. *Qual Health Res*. 2016;26(6):741-749. doi:10.1177/1049732316631510
6. Tuttas CA. Lessons learned using web conference technology for online focus group interviews. *Qual Health Res*. 2015;25(1):122-133. doi:10.1177/1049732314549602
7. Reid DJ, Reid FJM. Online Focus Groups: An In-depth Comparison of Computer-mediated and Conventional Focus Group Discussions: *https://doi.org/101177/147078530504700204*. 2018;47(2):131-162. doi:10.1177/147078530504700204
8. Underhill C, Olmsted MG. An Experimental Comparison of Computer-Mediated and Face-to-Face Focus Groups: *http://dx.doi.org/101177/0894439303256541*. 2016;21(4):506-512. doi:10.1177/0894439303256541
9. Glen FC, Baker H, Crabb DP, Crabb D. A qualitative investigation into patients’ views on visual field testing for glaucoma monitoring. *BMJ Open*. 2014;4:3996. doi:10.1136/bmjopen-2013
10. Muth DR, Neubauer AS, Klingenstein A, Schaller U, Priglinger SG, Hirneiß CW. What would an ‘ideal’ glaucoma examination be like? - A conjoint analysis of patients’ and physicians’ preferences. *Int Ophthalmol*. 2021;41(12):3911-3920. doi:10.1007/s10792-021-01960-5
11. Sood D, Sood I, Sood S, Kumar D, Sood R, Sood NN. Patients’ perception of glaucoma diagnosis practice: Results from a survey of glaucoma patients in North India. *Int Ophthalmol*. 2020;40(7):1797-1805. doi:10.1007/s10792-020-01349-w
12. Owsley C, McGwin G, Scilley K, Girkin CA, Phillips JM, Searcey K. Perceived Barriers to Care and Attitudes about Vision and Eye Care: Focus Groups with Older African Americans and Eye Care Providers. *Invest Ophthalmol Vis Sci*. 2006;47(7):2797-2802. doi:10.1167/IOVS.06-0107
13. Burr JM, Botello-Pinzon P, Takwoingi Y, et al. Surveillance for ocular hypertension: An evidence synthesis and economic evaluation. *Health Technol Assess (Rockv)*. 2012;16(29):1-266. doi:10.3310/hta16290
14. NHS. Digital transformation. https://www.england.nhs.uk/digitaltechnology/.
15. Glaucoma: diagnosis and management NICE guideline. Published online 2017. Accessed July 26, 2023. www.nice.org.uk/guidance/ng81
